# Nano-Graphite Prepared by Rapid Pulverization as Anode for Lithium-Ion Batteries

**DOI:** 10.3390/ma15155148

**Published:** 2022-07-25

**Authors:** Wei Liu, Kai Zong, Ying Li, Yonggui Deng, Arshad Hussain, Xingke Cai

**Affiliations:** 1 Institute for Advanced Study, Shenzhen University, Shenzhen 518060, China; 2017161099@email.szu.edu.cn (W.L.); greengingko981@163.com (K.Z.); 2060391015@email.szu.edu.cn (Y.L.); arshadhussain977@yahoo.com (A.H.); 2 College of Mechatronics and Control Engineering, Shenzhen University, Shenzhen 518060, China; den09292020@163.com

**Keywords:** nano-graphite, mechanical pulverization, lithium-ion batteries, anode, high rate

## Abstract

Reducing the particle size of active material is an effective solution to the poor rate performance of the lithium-ion battery. In this study, we proposed a facile strategy for the preparation of nano-graphite as an anode for a lithium-ion battery via the rapid mechanical pulverization method. It is the first time that diamond particle was selected as the medium to achieve high preparation efficiency and low energy consumption. The as-prepared nano-graphite with the size from 10 to 300 nm displays an intact structure and high specific surface area. The introduced oxygen atoms increased the wettability of nano-graphite electrode and lowered its polarization. The nano-graphite prepared from three hours of grinding shows an excellent reversible capacity of 191 mAh g^−1^, at a rate of 5 C, after 480 cycles, along with an increase of 86% in capacity, at 1 C, in comparison with pristine graphite. The highlight of this strategy is to optimize the current preparation method. The good electrochemical performance comes from the combined effect of nano-scale particle size, large specific surface area, and continuous mesopores.

## 1. Introduction

The lithium-ion batterie (LIB), as one of the most important energy storage devices that plays an indispensable role in the world of production and living, has various advantages, such as a high average output voltage and high energy density, and it is also environmentally friendly [1]. The intercalation/deintercalation of Li^+^ plays an essential role in the electric energy storage and release converted from the chemical energy stored in the compound, laying the solid technical foundation of LIBs [2]. The evaluation of the performance of LIBs is based on several important indicators, including charge and discharge capacity, cycle life, and rate performance. Graphite is considered to be the most ideal anode material in LIBs and is widely used in the commercial application due to its stable electrochemical performance and high theoretical specific capacity (~372 mAh g^−1^) [3]. However, the poor fast-charging ability of the graphite-based LIB impedes its further development, especially for the application in electric vehicles and electrical power grids.

Designing a porous structure and nanosizing the graphite-based anode material can effectively enhance the high-rate performance of LIBs, since this could shorten the Li^+^ migration distance. In the meantime, the contact area between the electrolyte and electrode could be increased [4]. Recent studies have mainly focused on the design of porous graphite to improve the intrinsic capability in fast charging. For example, Deng et al. reported that the porous graphite synthesized by nickel-catalyzed gasification exhibits a high capacity of 200 mAh g^−1^ at 5 C [5]. Deng et al. found that the porous graphite shows an excellent capacity retention of 81.4% of its first irreversible capacity after 1500 cycles at 5 C, and this is much higher than that of commercial graphite [6]. Despite these intriguing merits of porous graphite, the practical application is still limited by its complicated synthesis route.

In recent years, only a few studies have focused on nanosizing graphite into nano-graphite. This is mainly due to the fact that exfoliating along the plane of the layered material overcomes the van der Waals forces. Along the direction perpendicular to the plane of the layered material, it is necessary to break the cooperation between atoms in the plane of the layered material. For example, the shear force applied on graphite in the vertical direction is 28 times stronger than the van der Waals force to be overcome in the plane direction [7]. Nano-graphite usually provides more effective space and active sites for the storage of lithium ions, which facilitate the migration of lithium ions during the charge/discharge process [8]. The normal preparation methods for preparing nano-graphite have been established, including ball milling [9,10] and mechanical grinding [11], which impact and grind the graphite from top to bottom to refine the bulk material to nanoscale size. For example, Li et al. obtained nano-graphite powder with a particle size of 20–30 nm and a few-layered structure (3–5 layers) by using the ball-milling method. It should be pointed out that the exfoliation process took 40 h to obtain nano-graphite, consuming a lot of energy during the process [12]. Meanwhile, during the high-energy collision, the milling medium, such as steel balls and zirconia balls, will lower the purity of the product. Therefore, it is necessary to optimize the method to achieve both a high preparation efficiency and good quality of product.

In this paper, we report about the novel synthesis route and applicable use of nano-graphite as anode in LIBs, achieving high specific surface area, comparable areal capacities with commercial graphite, outstanding cycle stability, and high-rate performance. We propose a simple model for the Li-ion storage mechanism, and it accounts for the short diffusion path of lithium ions during the lithiation/delithiation process, which results in the high-rate performance of the nano-graphite electrode.

## 2. Materials and Methods

### 2.1. Fabrication of Nano-Graphite

Nano-graphite was prepared from natural flake graphite (1200 mesh, Shanghai Macklin Biochemical Co., Ltd., Shanghai, China) by using a rapid pulverization method with the introduction of diamond particles (80 mesh, Ai-zuan Novel Materials Co., Ltd., Shangqiu, China). The mass ratio between natural flake graphite and diamond particle was 1:1, the grinding speed was set to 2800 rpm, and the grinding time varied from one hour to three hours. The as-pulverized nano-graphite materials were dispersed into isopropanol solution (purity ≥ 99.5%, Shanghai Macklin Biochemical Co., Ltd., Shanghai, China) at a concentration of 10 mg mL^−^^1^. After that, the dispersion was centrifuged at 3000 rpm for 10 min, and the supernatant was taken to obtain high-quality nano-graphite dispersion with an average particle size of 300 nm. The nano-graphite materials prepared according to different grinding times were named as NG-x, where NG stands for nano-graphite, and x represents the grinding time (1, 2, and 3 h).

### 2.2. Structural Characterization

The prepared nano-graphite samples are systematically characterized by various measurements, including UV-visible spectroscopy (Agilent Cary 5000, Palo Alto, CA, USA), powder X-ray diffractometer (XRD, Bruker D8 Advance, Saarbrucken, Germany), Raman spectrometer (Renishaw InVia, Gloucestershire, UK), X-ray photoelectron spectroscopy (XPS, PHI Versa Probe II, Waltham, MA, USA), zeta potential and granular meter (Anton Paar, Litesizer™ 500, Shanghai, China), scanning electron microscopy (SEM, Hitachi SU8010, Tokio, Japan), surface area and pore size analyzer (Anton Paar, Nova touch Quanta chrome, Shanghai, China), and galvanostatic charge/discharge measurement (LAND, CT2001A, Wuhan, China).

### 2.3. Electrochemical Characterization

The lithium-ion semi-battery measurements were carried out by using a half-cell system in CR2032-type coin cells. Nano-graphite was mixed with super P and polyvinylidene fluoride (PVDF) binder to form slurry at the weight ratio of 8:1:1. The electrode was prepared by casting the slurry onto copper foil, using a doctor blade, and drying in a vacuum drying oven at 60 °C for 6 h. The active material loading content is about 0.8 mg cm^−^^2^. The electrode was cut into circular pieces, with a diameter of 12 mm for coin-cell testing. Li-ion batteries were assembled with lithium as the counter electrode. The electrolyte was 1.0 M LiPF_6_ in ethylene carbonate/diethyl carbonate (EC/DEC), with a volume ratio of 1:1, and polypropylene (PP) membrane was selected as the separator. The fabrication of coin cells is operated in the glove box, with an Ar atmosphere (H_2_O and O_2_ < 0.01 ppm). The lithium-ion batteries were cycled between 0.01 and 3 V, at a constant current density of 0.1 C on LAND CT2001A battery testing system at 25 degrees Celsius. Different current rates, ranging from 0.1 to 5 C, were also utilized to measure the electrochemical response.

## 3. Results and Discussion

**Scheme 1:**
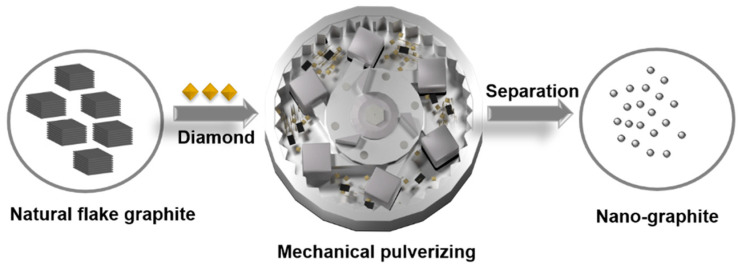
Three-dimensional schematic illustration of the pulverization process for the preparation of nano-graphite.

At present, various ways to prepare the nanoparticle have been established, including the mechanical pulverization and ball-milling method. Herein, we report a novel rapid pulverization method for the preparation of nanoparticle in this study (Scheme 1). The rapid exfoliation here is based on the shear force provided by the diamond perpendicular to the plane of the layered graphite flakes. The rotating grinding hammer in the cavity creates a strong wind field, which drives the mixture to move in an orderly manner at a high speed of 2000 rpm. With the synergistic effect of the centrifugal force and gravity, the grinding hammers collide to create a strong impact force perpendicular to the plane of graphite. The graphite flakes are pulverized on the collision surface, together with the collision with diamond particles. The shear force is applied in the direction of the plane of the graphite flakes to reduce the particle size. Since the size of the diamond particle is small, in the same grinding time, the particle size of the product obtained after the addition of the diamond is greatly reduced in comparison with the case of no addition.

**Figure 1:**
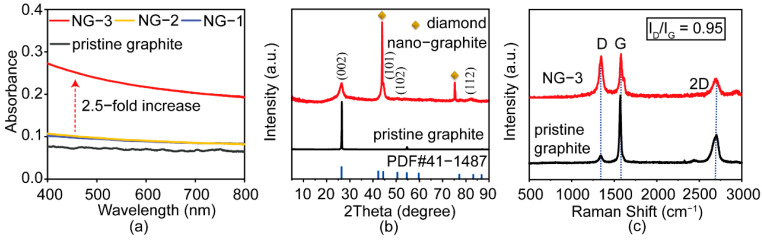
(**a**) UV–vis absorption spectrum for the supernate of pristine graphite and as-exfoliated NG-1, NG-2, and NG-3 after centrifugation. (**b**) XRD patterns and (**c**) Raman spectra of powder sample of pristine graphite and NG-3.

To characterize the as-obtained nano-graphite, three different nano-graphite materials from different grinding times, named as NG-1, NG-2, and NG-3, were dispersed in isopropanol (IPA) solvent, separated by centrifugation process, and re-dispersed in IPA. The stability of nano-graphite dispersion under centrifugation at 3000 rpm was checked after standing overnight. The quantitative analysis of the concentration difference was carried on by using UV–vis absorption spectrum. The intensity of the NG-3 curve in the range of 400–800 nm is 2.5-fold larger than the other curves, which implies that the concentration for NG-3 is also 2.5 times larger, correspondingly (Figure 1a). Therefore, NG-3 was selected for further characterization and electrochemical performance. The crystal structures of pristine graphite and as-prepared NG-3 were investigated by powder X-ray diffractometer (XRD) and Raman spectrometer. As shown in Figure 1b, both pristine graphite and as-prepared NG-3 display the characteristic peaks in the XRD pattern of NG-3 located at 2θ of 26.2 and 44.4 degrees, which come from the (002) and (101) crystal planes, respectively (Figure 1b). These two peaks are consistent with the characteristic peaks of graphite in 2H phase (PDF#41-1487) [13], which reveals that the crystal structure remains the same as natural flake graphite after the pulverization and refinement process. The other two peaks occur at 43.9 and 75.3 degrees and come from the non-well-separated diamond. It is worthy to notice that the board peak at 26.2 degrees for NG-3 is the evidence to show that the lateral size of nano-graphite is greatly cut down. Furthermore, the weak intensity and increased full width at half maximum (FWHM) of (002) plane of NG-3 indicate that the graphitization degree of the nano-graphite has decreased significantly [14]. A qualitative analysis for defects and disordered carbons was carried out based on Raman spectra (Figure 1c). Both pristine graphite and NG-3 show three characteristic peaks located at 1360 cm^−^^1^ (D band), 1580 cm^−^^1^ (G band), and 2695 cm^−^^1^ (2D band). The D band has been attributed to the in-plane C–C stretching A_1g_ vibration mode, which reflects the imperfect layers and disordered carbons. The G band corresponds to the splitting of the degenerate E_2g_ vibration mode [15]. The intensity ratio of D band and G band (I_D_/I_G_) determines the degree of imperfection (in-plane defects and edge defects) in the as-prepared nano-graphite [16]. The value of I_D_/I_G_ for NG-3 is calculated to be 0.95, and this result is 0.12 for pristine graphite. The increased value of I_D_/I_G_ indicates the rise of the imperfection degree for NG-3 after mechanical pulverization.

**Figure 2:**
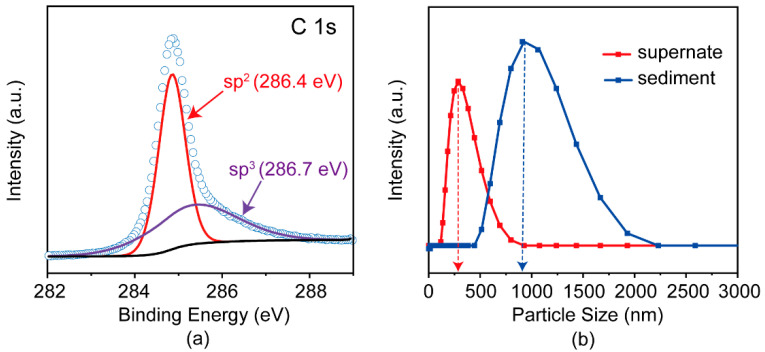
(**a**) XPS analysis of C 1s of the powder sample of NG-3. (**b**) Particle size distribution patterns of the supernate and sediment of NG-3 after centrifugation.

In addition, the element composition on the surface, as well as the existence state of nano-graphite material, was collected from XPS. The XPS result includes only C and O elements, in which the amount of oxygen is 9.85 at%. For raw graphite flakes, this value is 5 at%. According to peaks fitting of C 1s on Figure 2a, two different atomic bonding states of surface atoms are centered at 284.6 and 286.7 eV for sp^2^ and sp^3^ hybridization of carbon atom [17]. The introduction of oxygen atoms results from the high-energy pulverization process when carbon atoms combine with oxygen to form C=O and C–O bonds, effectively increasing the wettability of electrode materials and reducing the polarization of electrode materials. Meanwhile, oxygen atoms lead to uneven charge distribution of the material, and this is beneficial to the conduction of charges [18]. These characters are essential in the working process of LIBs.

**Figure 3:**
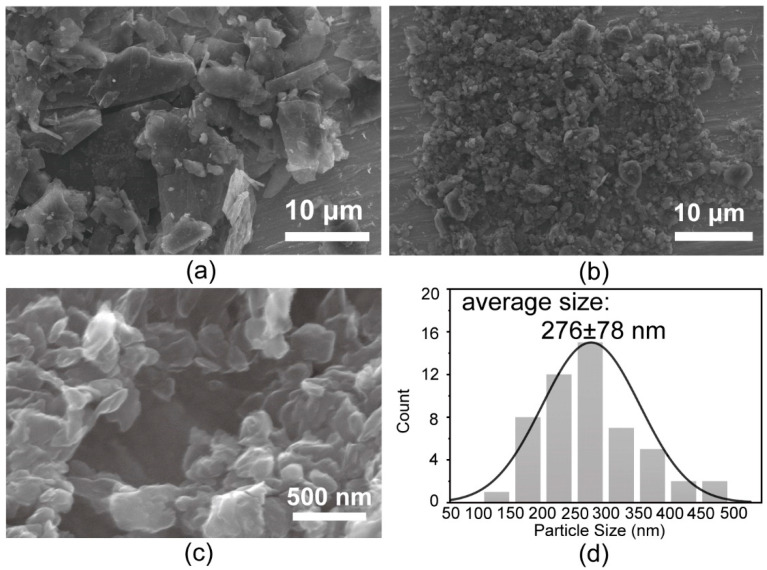
Characterization on the morphology and statistical analysis of particle size. (**a**) SEM image of pristine graphite flakes. (**b**,**c**) SEM images of NG-3 powder. (**d**) Gaussian curve approximation of particle-size-distribution plot based on the statistical counting of NG-3 particles appearing in (**c**).

Subsequently, the morphologies of as-obtained NG-3 and natural flake graphite were investigated by SEM. In Figure 3a,b, pristine graphite mainly exists in the form of flakes possessing the lateral size up to 12 μm with the smooth surface, as well as a clear layered structure. As shown in Figure 3c, after pulverizing, the particle size of the nano-graphite was significantly reduced; the statistical analysis of particle sizes is presented in Figure 3d. Fifty nano-graphite particles in Figure 3c participated in the statistics, and the bar graph demonstrates that the size of the majority of particles lies in the range of 250–300 nm, leading to the average size of 276 (±78) nm. A further visual result was presented from zeta potential and particle-size-analyzer based on DLS analysis (Figure 2b). The as-prepared NG-3 was separated into two parts after centrifugation at the speed of 3000 rpm, including the sediment and supernatant. The particle-size-distribution plot of the supernatant follows the Gaussian curve approximation, giving the most abundant particle sizes, ranging from 255 to 355 nm, and the average value, located at 295 nm. The blue curve represents the average particle size, up to 1 μm, coming from non-well-exfoliated graphite. The statistical analysis in the SEM image and the quantitative DLS analysis are two pieces of evidence that show the reduction of the particle size. During the pulverization process, the surface roughness and irregular edges increase because the shear force generated by the impact of the diamond and the effective collision with inner wall of the container damage the ordered structure of the graphite. In addition, the agglomeration of the nano-graphite particles ascribes to the higher specific surface energy of the particles.

**Figure 4:**
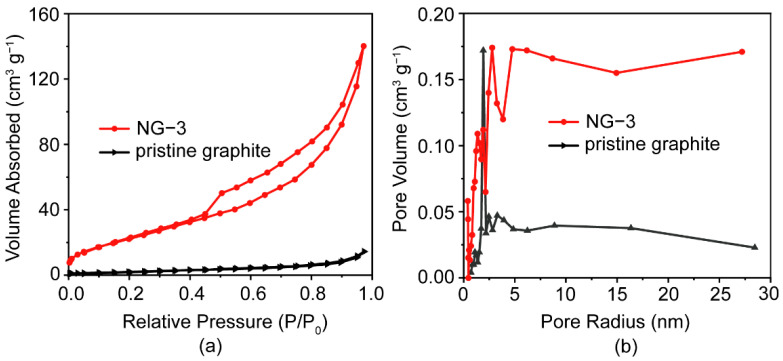
(**a**) The distribution plots of pore size and (**b**) isothermal N2 absorption for pristine graphite and NG-3 powder.

Figure 4 presents the surface area and pore size distribution based on the BET theory. According to the nitrogen adsorption-desorption curve, there is a significant increase of Brunauer–Emmett–Teller (BET) specific surface area for NG-3 (138 m^2^ g^−^^1^), as compared with pristine graphite (8 m^2^ g^−^^1^). Although the specific surface area of NG-3 is smaller than the theoretical value of mesoporous materials (~2000 m^2^ g^−^^1^), this value (138 m^2^ g^−^^1^) is similar to the those of previously reported graphene nanosheets (184 m^2^ g^−^^1^) [2]. In addition, as shown in Figure 4a, both the curves of NG-3 and pristine graphite show a hysteresis loop, corresponding to typical IV type of isothermal adsorption curve and the classification of H3, indicating that a large number of mesoporous channels are formed by material accumulation [19]. The pore size distribution plots in Figure 4b demonstrate that the average pore radius is about 2.2 nm for NG-3 and 1.8 nm for pristine graphite based on the BJH model; this further proves the existence of mesopores in NG-3. The increase of pore radius and uneven distribution of disordered mesopores of NG-3 mainly result from the shear force at random direction during the pulverization process and uneven distribution of generated defects. Moreover, the micropore volume is only 0.05 cm^3^ g^−^^1^, indicting a very low content of micropores.

**Figure 5:**
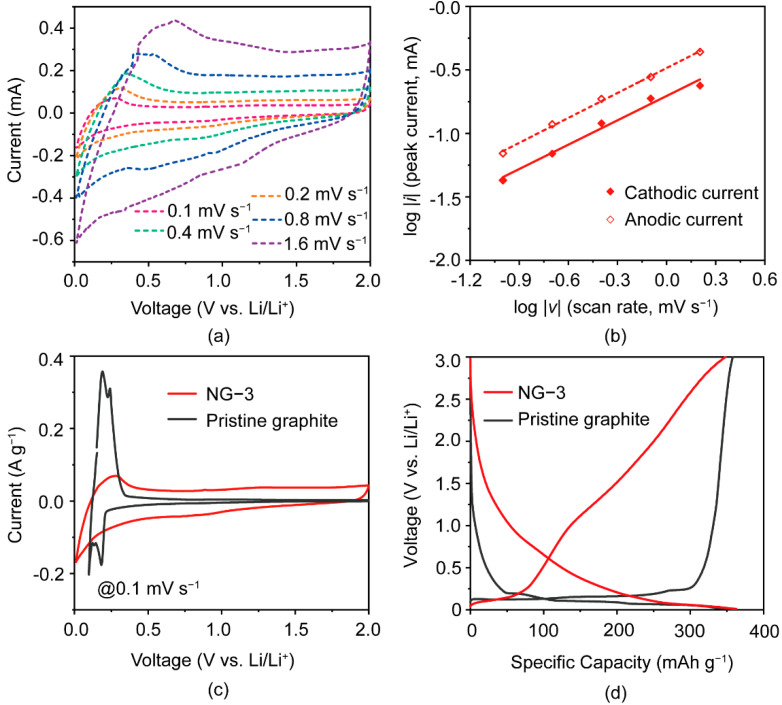
(**a**) Cyclic voltammetry curves of NG-3 electrode at a scan rate ranging from 0.1 to 1.6 mV s^−1^. (**b**) Log|v| − log|i| plot to determine b value based on its slope. (**c**) Cyclic voltammetry curves of NG-3 and natural graphite electrode at a scan rate of 0.1 mV s^−^^1^. (**d**) Galvanostatic lithiation/delithiation profiles of NG-3 and natural graphite electrode at current density of 0.1 C.

Galvanostatic charge–discharge techniques were utilized for the electrochemical performance of the as-obtained nano-graphite. In order to understand the rection kinetics of Li ions, we carried out the cyclic voltammetry experiments for NG-3 and natural graphite under different sweeping rates. As presented in Figure 5a, anodic and cathodic current peaks are located at 0.25 and 0.75 V, respectively, indicating the removal and extraction behavior of lithium [20]. In addition, the inconspicuous broad peak at 1.2 V mainly results from the reversible redox reaction that occurred from the deintercalation of Li^+^. We then consider the charge-storage mechanism by qualitatively analyzing the relationship in CV experiment between peak current, i, and scan rate, v: $i\left(v\right)=av^{b}$, where *a* is the constant, and *b* stands for the power-law exponent [21]. Experimentally, the *b* value is 0.5 and 1 for the battery material and pseudocapacitive material, corresponding to a diffusion-controlled and capacitor-like process. For NG-3 electrode, the *b*-values of anodic and cathodic current were calculated to be 0.65/0.64 based on the Figure 5a. The results of the *b*-value lie within 0.5 to 1 and are very close to 0.5, indicating a mixture of diffusion-controlled and capacitor-like process but with diffusion-controlled mechanism being dominate [22]. The CV curve of natural graphite displays defined anodic and cathodic peaks at around 0.2 V/0.1 V, thus suggesting its battery-like characteristic (Figure 5c). Both NG-3 and natural graphite show a similar anodic peak, and NG-3 exhibits a higher cathodic peak at 0.75 V as compared to natural graphite. Moreover, the Galvanostatic lithiation/delithiation profile of the NG-3 electrode is another way to tell the difference in the discharge voltage plateau (Figure 5d). The discharge voltage plateau (~0.1 V) for natural graphite is extremely low, and this will cause a safety problem due to the formation of lithium dendrites, especially at a high current density [23]. The rise of the discharge voltage plateau (~0.75 V) for the NG-3 electrode should improve the safety of the cell.

**Figure 6:**
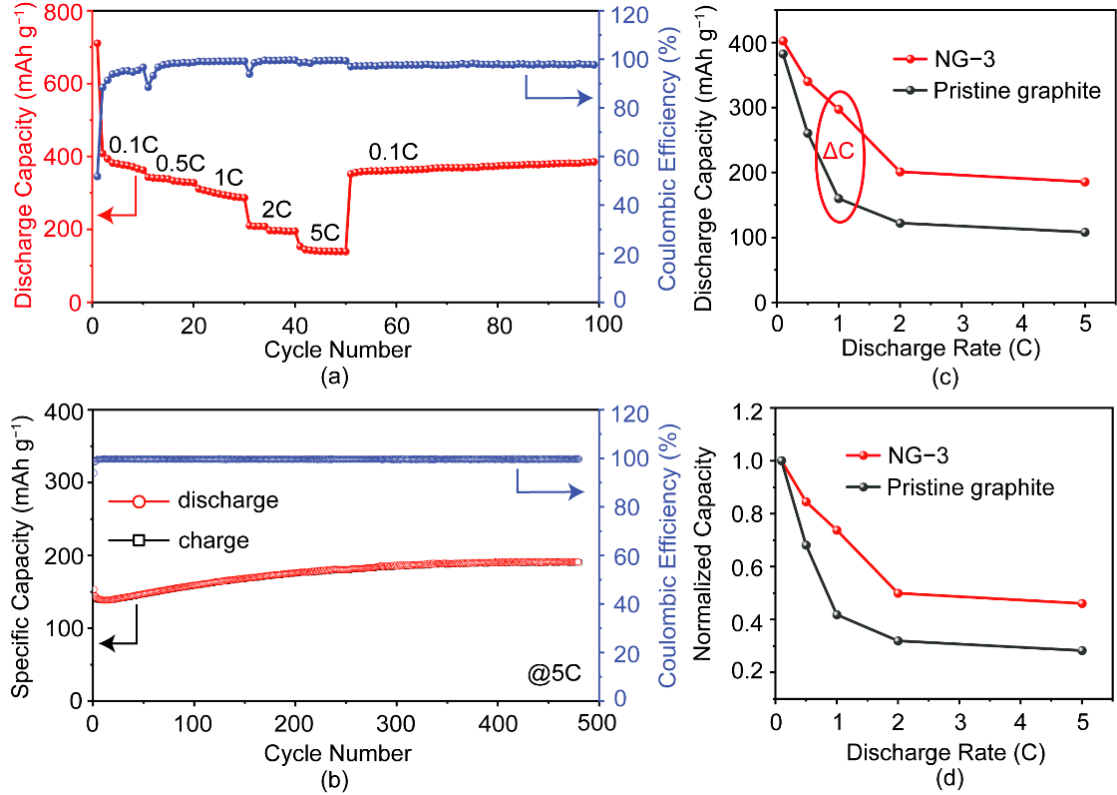
(**a**) Rate capability of discharge capacity and coulombic efficiency for NG-3 at various current rates between 0.01 and 3.0 V vs. Li/Li^+^. (**b**) The cycle stability of NG-3 at the rate of 5 C. (**c**) The capacity and (**d**) normalized ones at different current rates for pristine graphite and NG-3.

Furthermore, the rate performance shows that the discharge capacity varies as current densities increase for NG-3 (Figure 6a). The discharge capacities for NG-3 were measured to be 340.3 mAh g^−^^1^ at a rate of 0.5 C, 297.2 mAh g^−^^1^ at 1 C, 201.2 mAh g^−^^1^ at 2 C, and 185.5 mAh g^−^^1^ at 5 C. As the current density moved back to 0.1 C after 50 cycles, the NG-3 electrode could still deliver a capacity of 380 mAh g^−^^1^. In addition, NG-3 shows a stable circulation performance, with a good reversible capacity of 191 mAh g^−^^1^ after 480 cycles and stable coulombic efficiency around 100% (Figure 6b). The Galvanostatic lithiation/delithiation profiles of NG-3 electrodes under 5 C could be separated into three parts: (1) from the first to 20th cycle, the capacity decreases from 153.5 mAh g^−^^1^ to 139.5 mAh g^−^^1^; (2) negative fading happens from the 21st to the 350th cycle; and (3) stabilization starts from the 351st cycle, delivering a capacity of 191 mAh g^−^^1^. The negative fading can be mainly ascribed to the continuous consumption of lithium and electrolyte-derived surface reaction, leading to the increased capacity [24].

In contrast, the rate performance tests for commercial pristine graphite were also carried out. The comparison of discharge capacities between NG-3 and pristine graphite is presented in Figure 6c,d. It should be pointed out that the capacities of NG-3 are always greater than those of pristine graphite at any current densities. To be specific, the largest difference is calculated to be 86% (Δc = 137.2 mAh g^−^^1^) of increase at a rate of 1 C for NG-3, as compared with pristine graphite. The normalized capacities for NG-3 and pristine graphite are shown in Figure 6c. The capacities of both materials decline with the increase of current densities. Nevertheless, at a rate of 5 C, the capacity retention for NG-3 is 46%, while this result is only 28% for pristine graphite (Figure 6d). To be specific there was a dramatic improvement in results from the reduction of the particle size and the introduction of the porous structure, which significantly shortens the transport path of lithium ions between the nano-graphite sheets. In addition, the introduction of oxygen atoms on the surface of the nano-graphite also facilitates the migration of ions inside the electrode material.

## 4. Conclusions

A facile method to fabricate nano-graphite was developed by using a diamond-based pulverization technique wherein the diamonds were mixed with the natural graphite and then were cut to nanosize due to the force from the paddle of the crusher. The particle size of as-prepared nano-graphite fall into the range from 10 to 300 nm, with a high specific surface area of 138 m^2^ g^−^^1^. The nanosize of the graphite increases the contact area between the electrolyte and the electrode material, providing a shorter Li^+^ diffusion path inside the graphite. Consequently, the capacity at high current density increases. The NG-3 exhibits an excellent reversible capacity of 191 mAh g^−^^1^ at a high current rate of 5 C in LIBs, much higher than the natural graphite. Compared with the costly conventional method to prepare nano-graphite, the current method not only allows us to prepare them at a low cost at a large scale but also achieves high preparation efficiency and low energy consumption. The developed scalable method in this study should enable the nano-graphite to be a potential anode material for the next-generation high-rate LIBs.

## Data Availability

The data presented in this study are available upon request from the corresponding author.
